# Frailty in Chinese older adults: the roles of sedentary behavior, relative sit-to-stand power, and their additive interaction

**DOI:** 10.1186/s12877-026-07230-2

**Published:** 2026-02-26

**Authors:** Zhu Jiarong, Zhang Xu, Meng Qi, Wang Jingjing, Fan Chaoqun, Wu Yini, Wang Mengdie, Yang Yuting, Feng Qiang

**Affiliations:** National Physical Fitness and Scientific Fitness Research Center, China Institute of Sport Science, Beijing, 100010 China

**Keywords:** Frailty, Sedentary behavior, Relative sit-to-stand power, Additive interaction

## Abstract

**Background:**

Frailty is a critical geriatric syndrome. While sedentary behavior (SB) and low lower-limb muscle power, which can be efficiently assessed by the 30-second sit-to-stand (STS) test, are independent associated factors for frailty, their additive interaction, especially in Chinese older adults, remain unexplored.

**Objective:**

This study aimed to investigate the individual and additive interactions of SB and relative sit-to-stand (STS) power with frailty, and to explore urban-rural disparities in these relationships.

**Methods:**

This cross-sectional analysis used data from 13,686 community-dwelling older adults (aged 60–79) from the 2024 Annual National Physical Fitness Surveillance in China. Relative STS power was assessed using the 30-second STS test and the Alcazar equation, low relative STS power was defined as a value below 2.53 W/kg in men and below 2.01 W/kg in women. SB was measured via the Global Physical Activity Questionnaire. Frailty was defined using the Chinese FRAIL scale. Logistic regression was used to estimate odds ratios (ORs) and additive interactions were assessed using RERI (Relative Excess Risk due to Interaction) and AP (Attributable Proportion).

**Results:**

The prevalence of frailty was 20.4%. Both SB and low STS power were independently associated with higher frailty odds (per 1-hour SB increase: OR = 1.11, 95% CI: 1.09–1.14; per quintile decrease in STS power: OR = 1.08, 95% CI: 1.05–1.12). A significant additive interaction was found (RERI = 0.31, 95% CI: 0.02–0.59; AP = 0.17, 95% CI: 0.01–0.29), indicating that approximately 17% of the frailty prevalence in the study, can be attributed to the interaction between SB and low relative STS power. This interaction was significant in rural areas (AP = 0.33) but not in urban areas.

**Conclusion:**

Prolonged SB and low relative STS power jointly increase the prevalence of frailty in an additive manner among Chinese older adults, with a more pronounced effect in rural settings. Public health interventions should concurrently target reducing sedentary time and enhancing muscle power, with tailored strategies for urban and rural populations.

**Supplementary Information:**

The online version contains supplementary material available at 10.1186/s12877-026-07230-2.

## Introduction

Frailty is an age-related clinical syndrome characterized by increased vulnerability to stressors due to a decline in physiological reserves across multiple systems [1, 2]. It is highly prevalent among older adults and is associated with a heightened risk of adverse health outcomes, including disability, reduced quality of life, and premature mortality [3–5]. In China, large-scale national studies reveal that frailty affects 22.54% of community-dwelling older adults and is projected to reach 32.46% by 2030 [6], with an even higher prevalence (46.1%−51.2%) in the pre-frail state, and shows significant disparities across gender, rural-urban, and regional divides [7, 8]. Beyond its physical manifestations, frailty is strongly linked to a wide range of health domains such as cognitive decline, mental health disorders, unhealthy lifestyle behaviors, and poor physical fitness [9]. Understanding the multifaceted nature of frailty is essential for developing effective prevention and intervention strategies, particularly as global populations continue to age.

Identifying modifiable risk factors is essential for developing targeted interventions. Lower-body strength is a well-established predictor of frailty, and declining muscle power is closely associated with its progression. Relative muscle power—defined as muscle power (W) normalized to body mass (kg)—declines more rapidly than muscle mass, physical performance, and absolute strength [10–12], with reductions beginning after age 30 and accelerating significantly after age 65 [13]. Moreover, longitudinal studies have demonstrated that low muscle power predicts the onset of frailty as well as hospitalization and all-cause mortality in older adults [14, 15]. In these studies, relative muscle power was assessed using the 30-second sit-to-stand (STS) test and the Alcazar equation [16], enabling a simple, low-cost, and efficient evaluation in clinical settings. Compared to the five-repetition sit-to-stand test, the 30-second version offers wider applicability by allowing muscle power assessment even in individuals unable to complete five stands [17]. This makes the 30-second test particularly suitable for frailer populations and facilitates longitudinal monitoring of functional status. Importantly, cut-off values for low relative sit-to-stand power (2.53 W/kg for men and 2.01 W/kg for women) have been linked to higher odds of frailty and decline in activities of daily living [18, 19]. However, it remains unclear whether these thresholds are applicable to Chinese older adults.

SB, defined as any waking behavior characterized by an energy expenditure ≤ 1.5 METs while sitting, reclining, or lying, has emerged as a significant public health issue [20], particularly among older adults. The 2020 WHO guidelines on physical activity and sedentary behavior emphasize that limiting sedentary time is crucial for healthy aging, as excessive sitting is associated with adverse outcomes such as increased all-cause mortality [21], cardiovascular disease [22], type 2 diabetes [23], and poorer mental health [20]. In the context of frailty—a state of heightened vulnerability to stressors—SB may exacerbate declines in muscle strength, functional capacity, and overall physiological reserve, thereby accelerating the progression of frailty.

Despite growing recognition of its harms, most existing studies on SB and frailty have been conducted in Western populations [24–26], with limited evidence from low-and middle-income countries, including China. Moreover, Previous studies usually focused on the separate association of SB or relative STS power with the prevalence of frailty and have largely ignored their combined effect or interactions between behavioral and Health-related factors, particularly across diverse socioeconomic and environmental settings such as urban and rural areas. This study therefore aims to investigate the associations of SB and lower-limb strength with frailty among Chinese older adults, and to explore how these relationships may vary between urban and rural contexts. The findings will help inform targeted interventions to reduce sedentary time and promote muscle strength in this rapidly aging population.

## Methods

### Study population sampling

The National Physical Fitness Surveillance (NPFS) is a nationwide program led by the General Administration of Sport of China, designed to assessing the physical fitness across the entire population. As previously reported, the NPFS employs a multistage, stratified probability cluster sampling design to ensure national representativeness, conducting standardized testing across all 31 provinces, autonomous regions, and municipalities in mainland China. [27, 28] This study employs data from the 2024 annual National Physical Fitness Surveillance (ANPFS), a program began in 2023 to be conducted annually. The establishment of this routine survey addresses the limitation of the excessively long five-year interval inherent in the previous NPFS cycle [29].

The sampling framework for the ANPFS was structured into two main stages. First, representative monitoring sites were selected using the Mean of Surface with Non-homogeneity (MSN) method, which accounts for spatial heterogeneity and autocorrelation in physical fitness levels across China. The country was stratified into seven geographic regions to minimize within-stratum variance and maximize between-stratum differences. Semi-variograms were modeled for both within and between strata to inform the sampling strategy. Among several sampling scenarios evaluated, a total of 150 monitoring sites were ultimately selected, as this achieved a sampling error of approximately 0.0005 while balancing operational feasibility and cost. These sites were distributed across 26 provinces, covering eastern, central, western, and northeastern China.

**Fig. 1:**
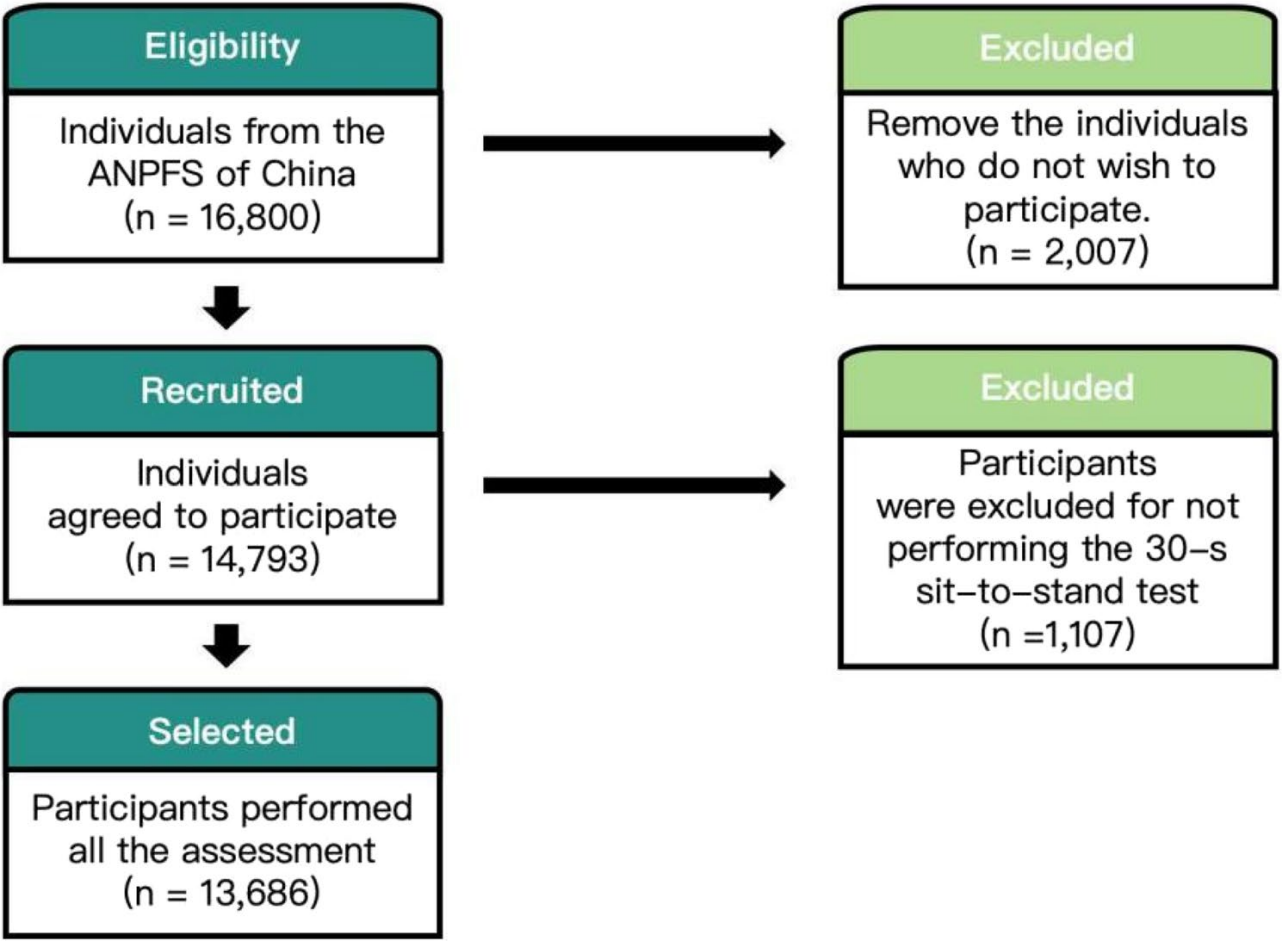
Flow chart of study participants

In the second stage, sample sizes were determined using a mean-based estimation approach, considering a design effect of 4, a 95% confidence level, and a relative error rate of 10% for indicators stratified by sex, age, and urban-rural residence. Across 150 monitoring sites, the target population was stratified into 16 groups by sex, age, and urban/rural residence, with 7 participants sampled from each group per site, resulting in a total national sample size of 16,800 participants. (Details in Supplementary File2) Eligible individuals from these sites were enrolled based on their willingness and ability to complete all tests, leading to a final sample size of 13,686 (Fig. 1). All the participants provided informed written consent, and the ANPFS study was granted ethics approval from the Ethical Review Committee of the China Institute of Sport Science and its collaborating institutions (GJTYZJ-2019021).

### Relative STS power and sedentary behavior measurement

Relative STS power was assessed using the 30-s STS test and the Alcazar Eq [30]. Participants were instructed to complete the maximum number of STS repetitions within 30 s after the cue “ready, set, go!” on a 0.43 m standardized chair without armrests. Participants had to perform the test with their arms crossed over their chest, and the STS repetitions were considered valid when the participant achieved a full standing position (full knee and hip extension) and at least touched the chair with their buttocks when sitting. The maximum number of repetitions completed in the 30-s STS test was recorded, and the Alcazar equation was used to estimate relative STS power: [30]

$$\mathrm{Relative}\;\mathrm{STS}\;\mathrm{power}\left(\frac{\mathrm W}{\mathrm{kg}}\right)=\left(0.9\times\mathrm g\right)\times\frac{\mathrm{Body}\;\mathrm{height}\;(\mathrm m)\times0.5-\mathrm{Chair}\;\mathrm{height}\left(\mathrm m\right)}{\left({\displaystyle\frac{30\mathrm s}{\mathrm{Number}\;\mathrm{of}\;\mathrm{STS}\;\mathrm{repetitions}}}\right)\times0.5}$$

Low relative STS power was defined as a value below 2.53 W/kg in men and below 2.01 W/kg in women, while the remaining participants were categorized as having high relative STS power. Participants unable to perform a single STS repetition were also classified as having low relative STS power. [31] We also converted relative STS power into a quintile-based continuous variable to examine the effect on frailty per one-quintile decrease.

Sedentary behavior was assessed through an interview using the Global Physical Activity Questionnaire (GPAQ) [32]. This assessment was conducted by trained interviewers at a mobile examination center using a computer-assisted personal interview system. Participants were asked: “How much time do you usually spend sitting on a typical day?” Specifically, SB was defined as the time spent sitting at school or at home and getting to and from places, including sitting at a desk, traveling in a car or bus, reading, playing cards, watching television, or using a computer, which do not include time spent sleeping. For the purpose of analysis, we classified participants reporting ≤ 4 h of daily sedentary time as having “low SB,” and those reporting > 4 h as having “prolonged/high SB.” This 4-hour threshold was determined a priori based on its clinical relevance and alignment with the distribution in our sample and previous studies in older populations [33].

### Outcome ascertainment

Frailty was assessed using the Chinese version of the FRAIL scale, a self-reported screening instrument comprising five components: fatigue, resistance, ambulation, illnesses, and loss of weight. Participants were asked whether they had experienced fatigue most or all of the time in the past month; whether they had difficulty climbing one flight of stairs or walking 100 m without assistance; whether they had been diagnosed with five or more chronic diseases from a specified list (e.g., hypertension, diabetes, asthma, arthritis, chronic lung disease, angina, acute heart attack, stroke, malignancy, or congestive heart failure); and whether they had experienced ≥ 5% weight loss within the past year. Each affirmative response was scored as 1 point. The total score ranged from 0 to 5, and participants were categorized as robust (0 points), pre-frail (1–2 points), or frail (≥ 3 points). The Chinese FRAIL scale has demonstrated satisfactory diagnostic accuracy against the Fried frailty phenotype, with an area under the curve of 0.91, and an optimal cut-point of 2 for identifying frailty [34, 35]. Internal consistency of the questionnaire was assessed using Cronbach’s alpha.

### Covariates

We presented the results of two statistical models. In the crude model, we only explored the exposure and outcome variables. In the adjusted model, we adjusted for age (60 to 79 years old), sex (male or female), region (rural or urban), education level (illiteracy, primary and junior high school, high school or above), household income (0-1k, 1k-5k, 5k-10k, > 10k), marital status (married, unmarried, divorced or widowed), living status (living alone or living with family), sleep quality (poor, rather poor, average, rather good or good), life satisfaction (poor, rather poor, average, rather good or good), alcohol status (yes or no), smoking status (yes or no), BMI (thin: <18.5 kg/m^2^, normal: 18.5–24 kg/m^2^, overweight: 24–28 kg/m^2^, obesity: >28 kg/m^2^). Based on the World Health Organization guidelines, older adults’ physical activity (PA) levels were classified as either “meeting recommendations” or “not meeting recommendations.” Participants were considered to meet the PA recommendation if they accumulated at least 150 min of moderate- to vigorous-intensity PA or at least 300 min of light-intensity PA per week. Those who did not achieve either of these thresholds were categorized as not meeting the recommendations.

### Statistical analyses

A generalized linear model (GLM) with binary logistic regression was used to evaluate the association between relative STS power, SB, and frailty, and to estimate odds ratios (ORs) with 95% confidence intervals (CIs). Relative STS power and SB were treated as separate categorical exposure variables. Additionally, SB was analyzed as a continuous variable, representing each additional hour. In this cross-sectional study, we used ORs to describe associations rather than risks, as temporal relationships could not be established.

Participants were classified into four groups based on relative STS power and SB levels: high relative STS power with low SB, high relative STS power with high SB, low relative STS power with low SB, and low relative STS power with high SB. Using the high relative STS power and low SB group as the reference, a GLM was applied in the adjusted model for OR analysis. Additive interaction was assessed using two indices: the relative excess risk due to interaction (RERI) and the attributable proportion due to interaction (AP) [36]. The 95% CIs for RERI and AP were generated by drawing 5,000 bootstrap samples from the estimation dataset [37]. If no additive interaction existed, the CIs for RERI and AP would include 0.

We conducted several sensitivity analyses to examine the robustness of the results. First, we included additional sedentary-related variables in the model, such as screen time. Second, we restricted the analysis to participants with complete covariate data to compare results with those obtained using multiple imputation [38]. Third, sedentary time was operationalized in multiple ways: as a continuous measure in hours and as a categorical variable defined by different thresholds (4, 6, 8, and 10 h). To assess the influence of pre-existing health conditions, we re-analyzed the data after excluding participants with self-reported chronic diseases. Additionally, the potential for unmeasured or uncontrolled confounding was evaluated using E-values. A large E-value indicates that considerable unmeasured confounding would be necessary to explain away an effect estimate, while a small E-value suggests that little unmeasured confounding would be needed to explain it away [39].

All P values were two-sided, and *P* < 0.05 was considered to indicate statistical significance. All analyses were performed by using R software, version 4.1.0 (R Foundation for Statistical Computing). Binary logistic regression and additive interaction were performed by using R packages, including the stats and interactionR packages.

## Result

### Study population characteristics

A total of 13,686 older adults (mean age 67.8 ± 5.4 years; 56.1% female) were included in this cross-sectional analysis. The overall prevalence of frailty was 20.4%. Several sociodemographic and lifestyle factors were associated with frailty status. Specifically, higher frailty rates were observed among participants who were divorced (32.9%), lived alone (28.4%), had lower household income (23.4% in the lowest income group), reported poor sleep quality (35.2%) or life satisfaction (46.3%), were smokers (31.4%), had low PA levels (24.8%), and the thin BMI category (27.6%). Notably, the low relative stand-to-sit power group showed a higher frailty prevalence (22.4%) compared to the high-power group (19.2%), and the high SB group had a substantially higher frailty rate (27.1%) than the low sedentary group (17.6%). The details are presented in Table 1.

**Table 1:** Population characteristics included in the study

Characteristic	*N*/%	Frailty rate/%
Total	13,686	20.
Sex		
Male	6012/43.9	20.9
Female	7674/56.1	20.0
Age, year, mean (SD)	67.8(5.4)	
Age group		
60–64	4448/32.5	20.8
65–69	3978/29.1	18.5
70–74	3222/23.5	21.7
75–79	2038/14.9	21.2
Area, %		
Rural	8812/64.4	19.9
Urban	4874/35.6	21.3
Education level, %		
Illiteracy	851/6.2	23.4
Primary and junior high school	7218/52.4	41.2
High school or above	5717/41.5	35.4
Marital status		
Married	12,538/91.6	19.5
Unmarried	153/1.1	21.6
Divorced	240/1.8	32.9
Widowed	755/5.5	19.5
Living status		
living alone	1513/11.1	28.4
living with family	12,173/88.9	19.4
Household income, %		
0-1k	851/6.2	23.4
1k-5k	7218/52.7	20.5
5k-10k	4811/35.2	20.2
>10k	806/5.9	17.1
Sleep quality, %		
Poor	776/5.7	35.2
Rather poor	319/2.3	40.1
Average	4833/35.3	24.8
Rather good	4413/32.2	16.0
Good	3345/24.4	14.6
Life satisfaction, %		
Poor	378/2.8	46.3
Rather poor	1045/7.6	24.7
Average	1386/10.1	31.1
Rather good	8375/61.2	18.6
Good	2502/18.3	14.7
Alcohol status, %		
Yes	2064/15.1	18.4
No	11,622/84.9	31.4
Smoking status, %		
Yes	1948/14.2	33.5
No	11,738/85.8	18.2
Physical activity level, %		
Low	9818/71.7	18.7
High	3868/28.3	24.8
BMI, kg/m^2^, mean (SD)	24.5(3.2)	
BMI, %		
Thin (< 18.5 kg/m^2^)	330/2.4	27.6
Normal (18.5–24 kg/m^2^)	5813/42.5	19.4
Overweight (24–28 kg/m^2^)	1775/13.0	20.4
Obesity (> 28 kg/m^2^)	5761/42.1	22.3
Relative stand-to-sit power, %		
Low	5193/37.9	22.4
High	8493/62.1	19.2
Sedentary behavior, %		
Low	9647/70.5	17.6
High	4039/29.5	27.1

Abbs: *SD* Standard deviation

### Associations of SB and relative STS power with frailty

Longer sedentary time was consistently associated with higher odds of frailty. In the fully adjusted model, each additional hour of daily SB was associated with an 11% increase in the odds of frailty (OR, 1.11; 95% CI, 1.09–1.14). When analyzed using different thresholds, a significant dose-response relationship was evident. For instance, compared to less sedentary (≤ 4 h of sedentary time per day) individuals, those with sedentary time exceeding 6 h and 10 h per day had approximately a twofold (OR, 1.99; 95% CI, 1.73–2.29) and a 2.5-fold (OR, 2.56; 95% CI, 1.62–4.01) higher odds of frailty, respectively (Table 2).

**Table 2:** Associations of the odds of frailty and sedentary behavior

Cut-off Value	Crude Model		Adjusted Model	
	OR (95%CI)	*P* Value	OR (95%CI)	*P* Value
4 h	1.73 (1.59, 1.89)	< 0.001	1.59 (1.44, 1.74)	< 0.001
6 h	2.24 (1.07, 2.55)	< 0.001	1.99 (1.73, 2.29)	< 0.001
8 h	1.72 (1.37, 2.15)	< 0.001	1.33 (1.04, 1.70)	0.02
10 h	2.73 (1.79, 4.13)	< 0.001	2.56 (1.62, 4.01)	< 0.001
Per 1 h	1.15 (1.13, 1.18)	< 0.001	1.11 (1.09, 1.14)	< 0.001

Similarly, lower relative stand-to-sit (STS) power was significantly associated with an increased prevalence of frailty. When treated as a continuous variable, each quintile decrease in relative STS power (W/kg) was associated with an 8% increase in the odds of frailty in the adjusted model (OR, 1.08; 95% CI, 1.05–1.12). The association remained significant when STS power was analyzed as a categorical variable (low vs. high), with the lower power group having 12% higher odds of frailty (OR, 1.12; 95% CI, 1.01–1.23) (Table E2). 

### Joint associations and subgroup analysis

**Fig. 2:**
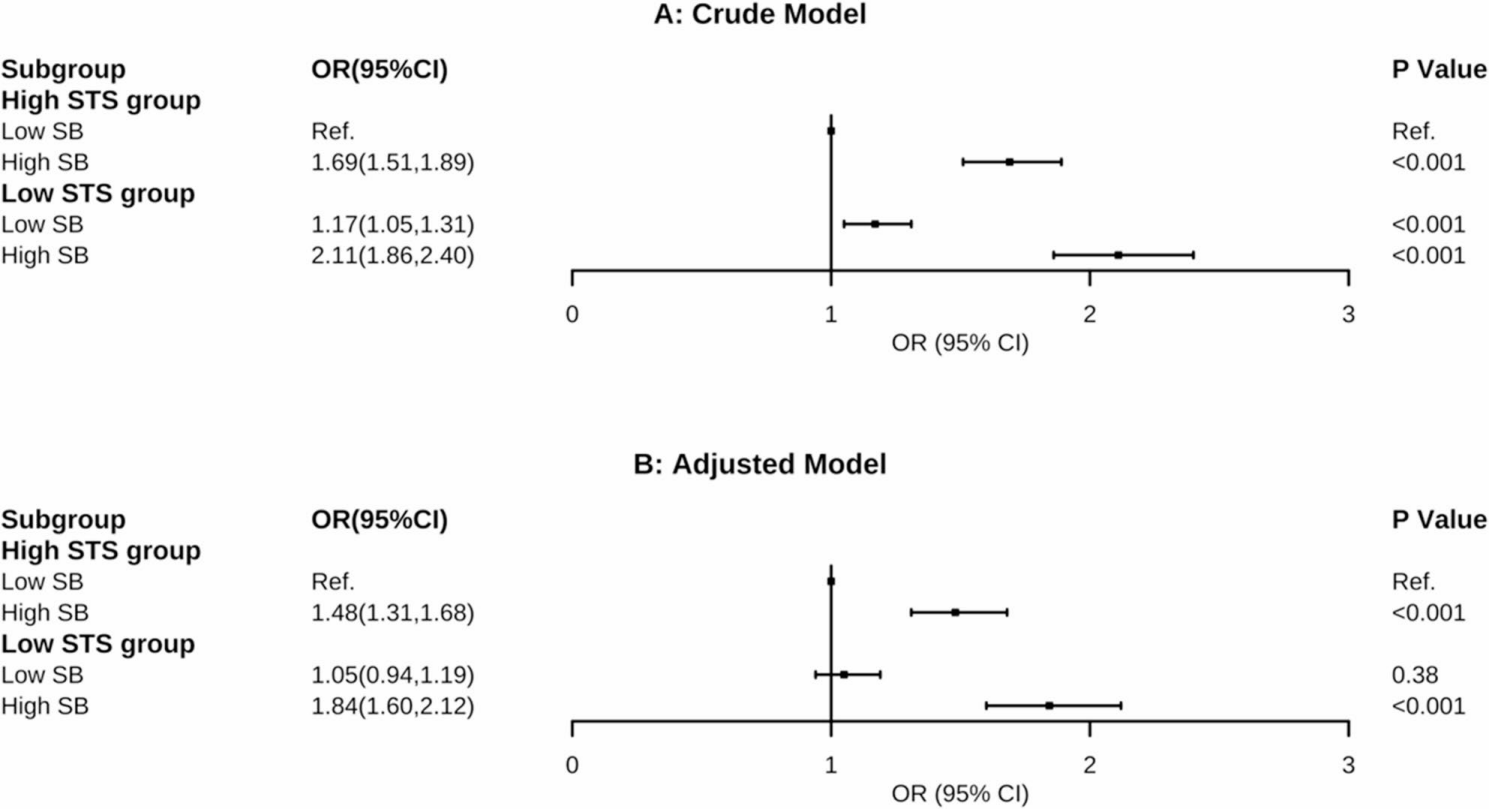
Odds of incident frailty according to sedentary behavior and relative stand-to-sit power categories. Abbs: OR = Odds Ratio; Ref = Reference; 95%CI = 95% Confidence

The joint associations of relative STS power and SB with frailty are presented in Fig. 2. In the adjusted model, compared to the reference group (high STS power/low SB), participants in the high STS power/high SB group had 48% higher odds of frailty (OR, 1.48; 95% CI, 1.31–1.68). Notably, those in the low STS power/high SB group showed the strongest association, with 84% increased odds of frailty (OR, 1.84; 95% CI, 1.60–2.12). However, in the low STS power group, individuals with low SB did not show significantly increased the odds of frailty compared to the reference group (OR, 1.05; 95% CI, 0.94–1.19; *P* = 0.38).

In the overall population (Table 3), a significant positive additive interaction was observed, with a relative excess risk due to interaction (RERI) of 0.31 (95% CI: 0.02, 0.59) and an attributable proportion due to interaction (AP) of 0.17 (95% CI: 0.01, 0.29). This indicates that 17% of the odds in the jointly exposed group (low STS power/high SB) was attributable to the interaction between these two factors. When stratified by area of residence, the additive interaction remained significant in rural areas (RERI: 0.60, 95% CI: 0.27, 0.95; AP: 0.33, 95% CI: 0.15, 0.46) but was not significant in urban areas (RERI: −0.25, 95% CI: −0.82, 0.29; AP: −0.13, 95% CI: −0.49, 0.12) details see (Table E6, E7).

**Table 3:** RERI and AP for additive interaction between sedentary behavior and relative stand-to-sit power categories

Total	RERI (95%CI)	AP (95%CI)	S (95%CI)
	0.31 (0.02, 0.59)	0.17 (0.01, 0.29)	1.57 (1, 2.46)
Rural Area	0.60 (0.27, 0.95)	0.33 (0.15, 0.46)	3.62 (1.17, 11.17)
Urban Area	−0.25 (−0.82, 0.29)	−0.13 (−0.49, 0.12)	0.78 (0.46, 1.32)

### Sensitivity analysis

The robustness of the primary findings was confirmed through several sensitivity analyses (see Supplement). In the sensitivity analyses, further adjustment for screen time did not significantly alter the associations with frailty (Supplement Table E3). We noted no significant differences in the ORs for having frailty before and after the exclusion of participants with incomplete covariate data. (Supplement Table E4). Sensitivity analysis yielded an E-value of 1.83. This indicates that an unmeasured confounder would need to be associated with both the exposure and the outcome with risk ratios of 1.83 each to fully explain the observed association. Compared to the measured confounders in this study, the strongest association was observed for smoking level (OR = 1.67), which is lower than 1.83, suggesting that the results are robust [39]. The FRAIL questionnaire demonstrated acceptable internal consistency (Cronbach’s α = 0.87).

## Discussion

This study provides a detailed examination of the complex relationship between SB (SB) and relative stand-to-sit (STS) power, two key associated factors for frailty in older adults. The main finding is that, among community-dwelling older adults, prolonged SB and low relative STS power exhibit a significant additive interactive effect on frailty, meaning their combined impact exceeds the sum of their individual effects. Importantly, the observed additive interaction was robust across multiple sensitivity analyses, including E-value calculations, suggesting that a relatively strong unmeasured confounder would be required to fully explain away the observed associations. Nevertheless, residual confounding cannot be completely ruled out, and causal interpretations should therefore be made with caution.

First, this study confirms that prolonged SB is a strong independent associated factor for frailty, for every additional hour of SB, the odds of frailty increases by 12%. Numerous previous studies in high-income developed countries have demonstrated that increased sedentary time in older adults is associated with a range of adverse health outcomes, including frailty [25], all-cause mortality [21], cardiovascular disease [22], type 2 diabetes [23], and poorer mental health [20], which is largely consistent with the findings of the present study. However, the molecular mechanisms by which SB contributes to chronic conditions remain unclear. The underlying mechanisms may involve that SB directly reduces muscle contraction, lowers energy metabolism, and may exacerbate insulin resistance [40] and chronic low-grade inflammation [41]. Physical inactivity and SB are independent but related risk factors [42]; even when PA guidelines are met, prolonged sedentary time still increases disease risk [43]. Moreover, one study has shown that reducing sedentary time may be more effective for improving glycemic control than increasing structured PA alone [44]. Therefore, for older adults, reducing SB in addition to increasing PA represents a more efficient and scientifically supported strategy to reduce frailty and improve health.

We conducted multi-threshold classification of SB. A slight attenuation in the association was observed at the 8-hour threshold (adjusted OR = 1.33), followed by a sharp increase in odds at the 10-hour threshold (OR = 2.56). This non-linear pattern may reflect population heterogeneity. This non-linear pattern may reflect population heterogeneity. Specifically, individuals with approximately 8 h of daily sedentary time may represent a heterogeneous group characterized by relatively preserved functional status and diverse lifestyle patterns, rather than a uniformly high-risk population. In contrast, those with 10 h or more likely represent a higher-prevalence subgroup, whose sedentariness may be more closely associated with poor health status (e.g., pain [45], functional limitations [2, 46], which possess anti-inflammatory and metabolism-enhancing properties that could partly counteract the chronic inflammatory state induced by SB. From a neuromuscular perspective, muscle power reflects the speed and efficiency of neural recruitment [47]; superior neuromuscular function in individuals with high power likely contributes to better balance [48] and fall resistance [49, 50], which are critical components in combating frailty.

This study provides an in-depth analysis of the complex relationship between SB and relative STS power, two critical associated factors for frailty in older adults. Most importantly, we are the first to identify an additive interaction between SB and relative STS power in relation to frailty. The results indicate that 17% (AP = 0.17) of the odds in the overall population is attributable to this additive interaction. This finding carries substantial clinical and public health implications, suggesting that older adults with the “high sedentary–low power” phenotype represent the highest-prevalence group for frailty. Biologically, this additive interaction may arise from SB and low muscle power accelerating frailty through distinct yet complementary pathways: sedentarism primarily acts at the systemic metabolic level (e.g., promoting inflammation [41] and insulin resistance [51]. Consequently, when individuals reduce participation in productive physical activities due to declining muscle power, they may struggle to find alternative light to moderate-intensity activities. This can lead to entrenched SB and a substantially elevated prevalence of frailty. The pattern of results in urban older adults is more complex. SB emerged as a powerful independent associated factor for frailty, with an effect size so substantial that it appeared to overshadow the contribution of low lower-limb muscle power. One plausible explanation is that in urban environments, SB (e.g., watching television, computer use, social sitting) constitutes a dominant, ingrained behavioral pattern. It is often closely linked to lifestyle choices rather than being primarily a consequence of declining physical function. Future research should aim to explain the specific mechanisms through which indicator contributes to frailty in different living environments.

Strengths of our study included: (1) The study leverages a large-scale, national surveillance dataset with a robust, multi-stage stratified sampling design, ensuring high generalizability to the Chinese older adult population. (2) This is one of the first studies to quantitatively examine the additive interaction between a behavioral factor (SB) and a physical fitness measure (relative STS power) on frailty, providing deeper insights beyond individual associations. (3) The use of the 30-second STS test and the Alcazar equation offers a simple, low-cost, and clinically feasible method for assessing a key physiological associated factor, enhancing the practical translatability of the findings. (4) The analysis provides a nuanced understanding of how the relationships between SB, muscle power, and frailty differ between urban and rural environments, highlighting the need for context-specific interventions. (5) The study employed comprehensive adjustment for confounders, multiple sensitivity analyses (including E-value calculation), and formal tests for additive interaction, which collectively strengthen the validity and robustness of the results.

## Limitation

Several limitations should be considered. First, the cross-sectional design precludes causal inference. Second, SB and PA were self-reported, which may introduce recall bias. Third, although multiple confounders were adjusted for, residual confounding from unmeasured factors (e.g., dietary habits, hearing/vision impairment, specific health conditions) remains possible. Fourth, the inclusion criterion requiring participants to be “willing and able to complete all physical tests” may have systematically excluded the frailest or functionally impaired older adults, introducing a potential selection bias. Fifth, there is a potential for conceptual overlap between the assessment of lower-limb muscle power (via the STS test) and certain components of the frailty outcome (e.g., resistance and ambulation in the FRAIL scale), which may influence the observed associations. Consequently, our findings may be less generalizable to the entire spectrum of frailty severity, particularly its most advanced stages. Finally, the generalizability of findings to institutionalized or older adults with severe disabilities may be limited, as the study included community-dwelling individuals.

## Conclusion

In conclusion, this study suggests that prolonged SB and low relative sit-to-stand power are independently associated with frailty among Chinese older adults, and that their co-occurrence is linked to a higher frailty burden, consistent with a positive additive interaction. This pattern was more pronounced in rural populations, highlighting potential contextual differences in the clustering of behavioral and physical risk factors. However, given the observational nature of the study and the possibility of residual confounding, these findings should be interpreted with appropriate caution. Nevertheless, the results underscore the potential value of jointly addressing SB and lower-limb muscle power when identifying high-risk groups and informing context-specific prevention strategies for frailty in older adults. 

## Supplementary Information

Supplementary Material 1.

Supplementary Material 2.

## Data Availability

The data that support the findings of this study were obtained from the Annual National Physical Fitness Surveillance, but restrictions apply to the availability of these data, which were used under license for the current study, and so are not publicly available.

## Abbreviations

SB: Sedentary Behavior
Relative STS Power: Relative Sit-to-stand Power
ORs: Odds ratios
NPFS: National Physical Fitness Surveillance
ANPFS: Annual National Physical Fitness Surveillance
MSN: Mean of Surface with Non-homogeneity
GPAQ: Global Physical Activity Questionnaire
GLM: Generalized linear model
CIs: Confidence intervals
RERI: The relative excess risk due to the interaction
AP: The attributable proportion due to the interaction
