# Supplementary material for: Frailty in Chinese older adults: the roles of sedentary behavior, relative sit-to-stand power, and their additive interaction
Source: BMC Geriatr. 2026 Feb 26;26:450. doi: 10.1186/s12877-026-07230-2 (PMC13040964; doi:10.1186/s12877-026-07230-2)
Supplement: Supplementary file 1 — Supplementary Material 1. [file 12877_2026_7230_MOESM1_ESM.docx]

# Supplementary files 2.

**Spatial Pattern of Physical Fitness in China**

A national map of physical fitness levels was generated through spline interpolation of comprehensive fitness scores obtained from the 2020 National Physical Fitness Survey (Figure 1). Stratification by geographic region (Northeast, North, Central, South, East, Northwest, and Southwest China) and subsequent geographical detector analysis revealed a q-value of 0.21 (*P* < 0.05), indicating significant spatial heterogeneity in physical fitness across China.


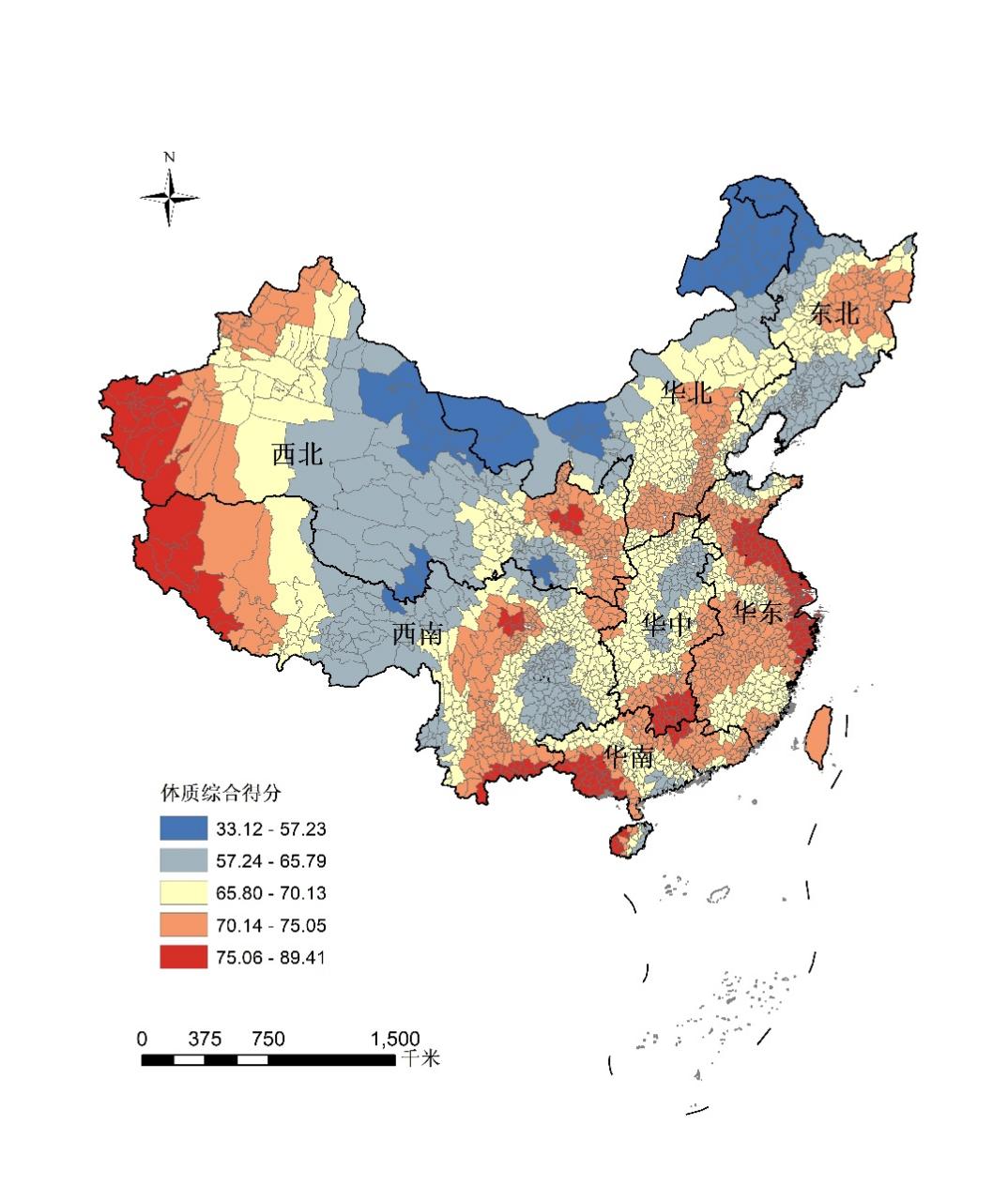


Figure 1 Spatial distribution of physical fitness levels in China

**Determination of Sampling Site Quantity**

Semi-variograms were calculated both between and within strata to evaluate spatial pattern differences and to inform the Mean of Surface with Non-homogeneity (MSN) sampling approach. The MSN method estimates the mean of regional variables exhibiting both auto-correlation and heterogeneity by stratifying heterogeneous areas into homogeneous subregions, estimating means for each subregion, and computing a weighted sum of these means to derive the overall estimate. Compared to conventional methods, MSN sampling integrates information within and between strata, achieving target sampling errors with smaller sample sizes while providing spatial locations of sampling points^1^.

Figure 2 demonstrates that geographical stratification effectively reduced within-stratum variance, with the best semi-variogram fits observed in East, South, and Southwest China, indicating lower estimation errors in these regions during sampling.


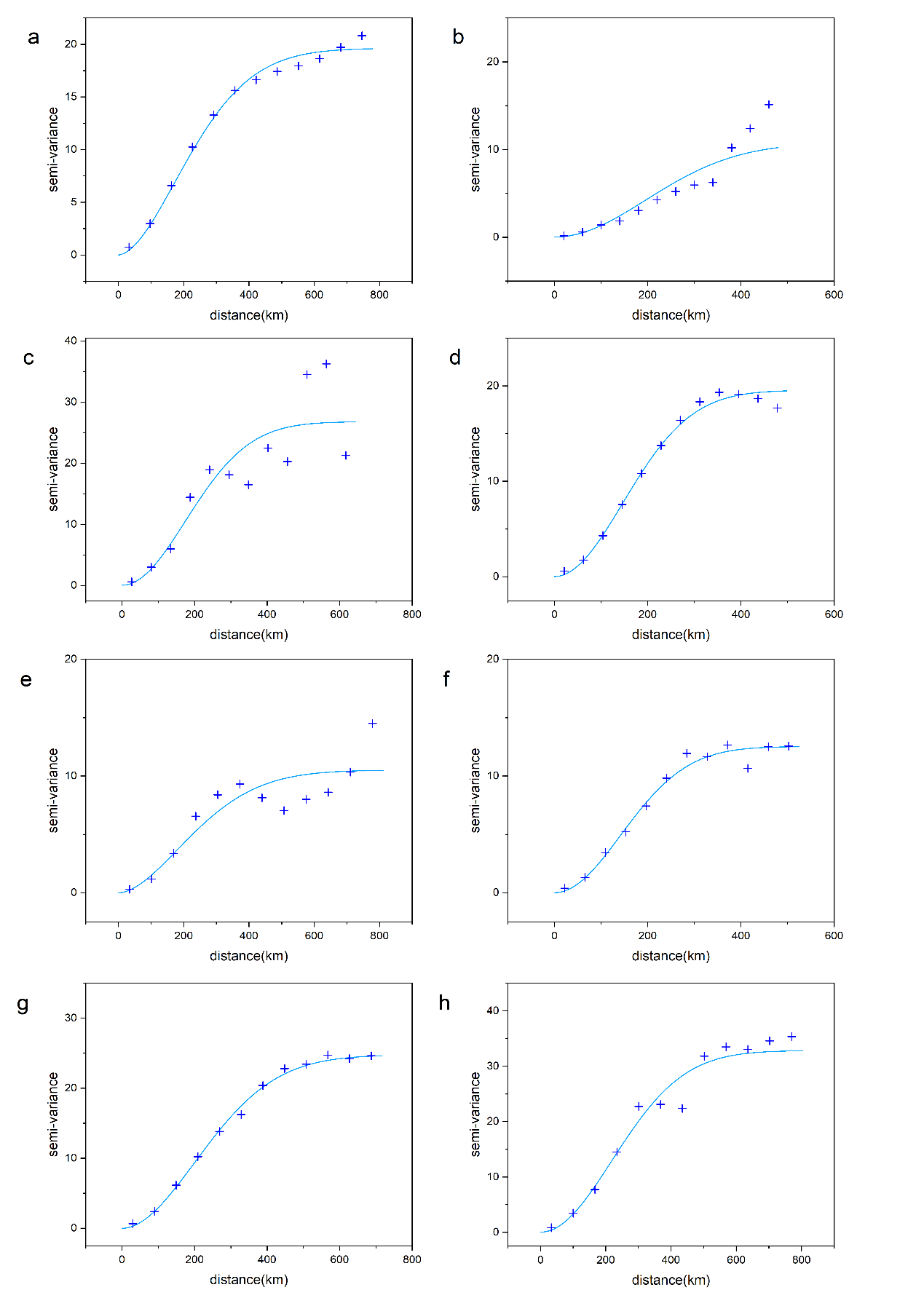


Figure 2 Semi-variograms between and within strata: (a) Between strata; (b) North China; (c) Northeast China; (d) East China; (e) Central China; (f) South China; (g) Southwest China; (h) Northwest China

Using the MSN approach, sampling errors were calculated for five sampling scenarios with sizes of 50, 100, 150, 200, and 250 sites (Figure 3). The sampling error decreased progressively with increasing sample size, approaching 0.0005 at 150 sites. Considering that this error margin met precision requirements and accounting for substantially increased costs and operational challenges with larger samples, a final sample size of 150 sites was selected.


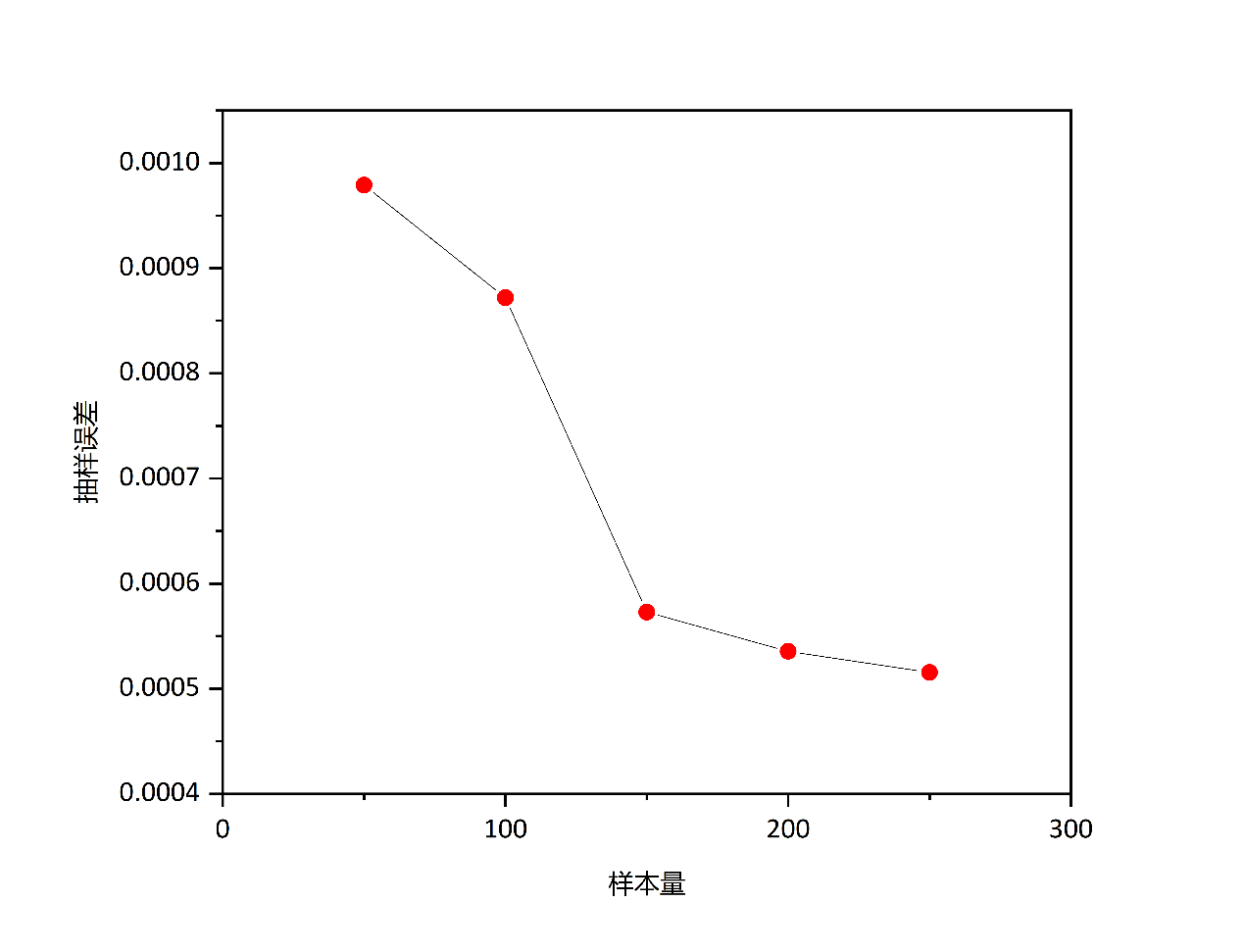


Figure 3 Sampling errors under different sample sizes

Figure 4 shows the spatial distribution of the 150 monitoring sites across 26 provinces/autonomous regions/municipalities. Geographically, these include: 8 in eastern China (Beijing, Hebei, Shanghai, Jiangsu, Zhejiang, Fujian, Shandong, Guangdong), 6 in central China (Shanxi, Anhui, Jiangxi, Henan, Hubei, Hunan), 10 in western China (Inner Mongolia, Guangxi, Chongqing, Guizhou, Yunnan, Shaanxi, Gansu, Qinghai, Ningxia, Xinjiang), and 2 in northeastern China (Liaoning, Heilongjiang).


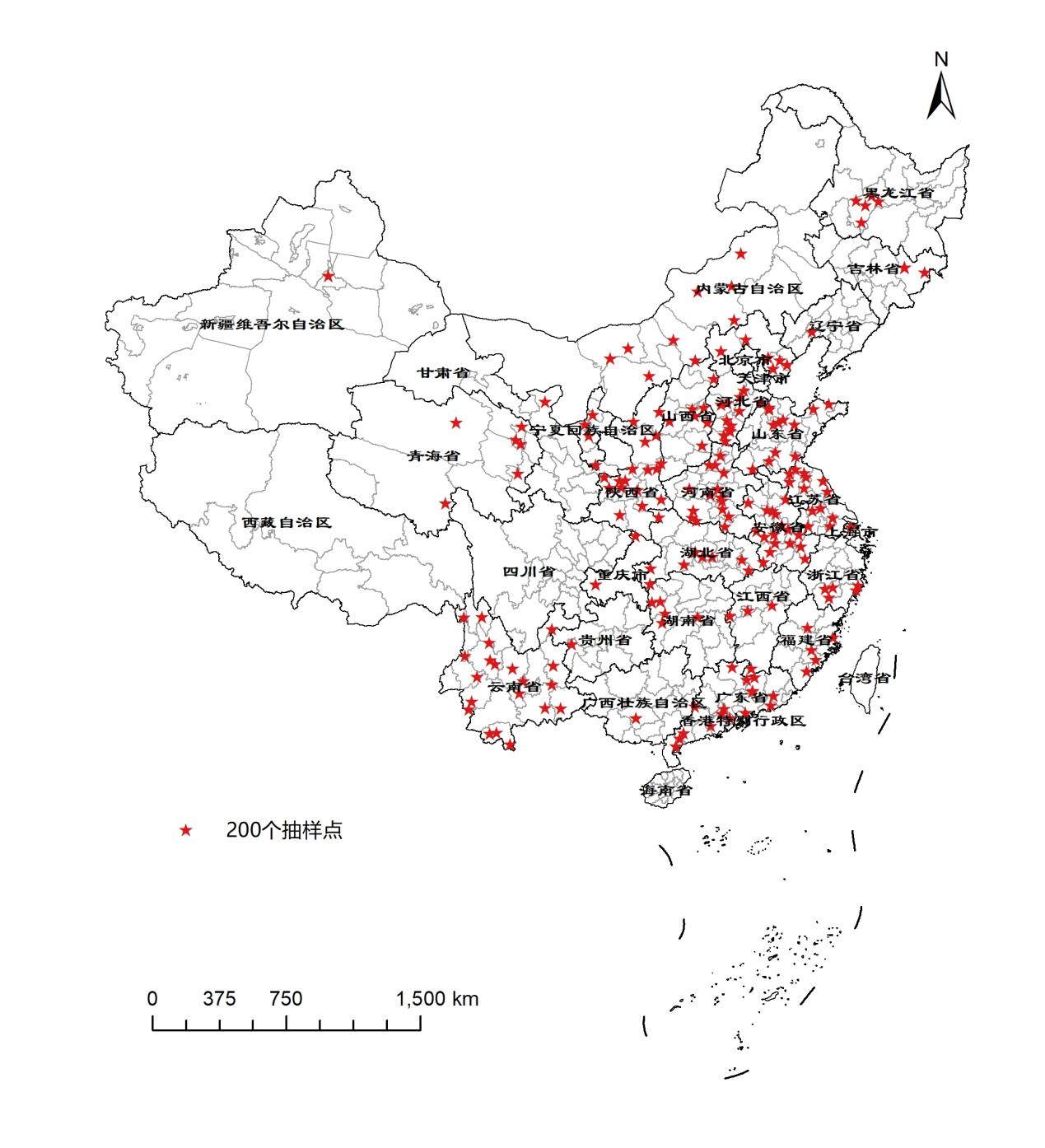


Figure 4 Spatial distribution of sampling sites

****Sample Size Determination****

Table 1 presents the estimated sample sizes for the physical fitness index (PFI) across subgroups defined by urban/rural residence, sex, and age. As a nationwide periodic survey, the sample size determination required comprehensive consideration of sampling efficiency, cost, budget, and completion rate. As shown in Table 1, the variability observed among older rural women was higher than in other subgroups. Basing the overall sample size calculation solely on this subgroup would have led to excessive workload demands. Therefore, after thorough evaluation, the following sampling scheme was adopted: across 150 monitoring sites, the target population was stratified into 16 groups by sex, age, and urban/rural residence, with 7 participants sampled from each group per site, resulting in a total national sample size of 16,800 participants.

**Table 1 Estimated Sample Sizes of the Physical Fitness Index by Urban and Rural Areas, Gender and Age Group**

| Physical fitness index | Male | | | | | | | | Female | | | | | | | |
| --- | --- | --- | --- | --- | --- | --- | --- | --- | --- | --- | --- | --- | --- | --- | --- | --- |
|  | Rural | | | | Urban | | | | Rural | | | | Urban | | | |
|  | M | SD | CV | n | M | SD | CV | n | M | SD | CV | n | M | SD | CV | n |
| 60-64 group | 67.2 | 6.55 | 0.10 | 7 | 70.4 | 6.29 | 0.09 | 4 | 65.7 | 7.40 | 0.11 | 8 | 69.8 | 7.23 | 0.10 | 5 |
| 65-69 group | 67.8 | 6.10 | 0.09 | 6 | 70.7 | 5.90 | 0.08 | 5 | 65.2 | 7.35 | 0.11 | 9 | 71.0 | 6.46 | 0.09 | 6 |
| 70-74 group | 67.4 | 6.60 | 0.10 | 8 | 70.7 | 6.29 | 0.09 | 7 | 65.4 | 7.60 | 0.12 | 9 | 69.5 | 6.25 | 0.09 | 9 |
| 75-79 group | 67.2 | 6.68 | 0.10 | 9 | 71.0 | 5.54 | 0.08 | 13 | 66.3 | 8.16 | 0.12 | 9 | 69.9 | 6.12 | 0.09 | 13 |

1 Jin-Feng Wang, Christakos G, Mao-Gui Hu. Modeling Spatial Means of Surfaces With Stratified Nonhomogeneity. *IEEE Trans Geosci Remote Sensing* 2009; **47**: 4167–74.
