# Supplementary material for: Frailty in Chinese older adults: the roles of sedentary behavior, relative sit-to-stand power, and their additive interaction
Source: BMC Geriatr. 2026 Feb 26;26:450. doi: 10.1186/s12877-026-07230-2 (PMC13040964; doi:10.1186/s12877-026-07230-2)
Supplement: Supplementary file 2 — Supplementary Material 2. [file 12877_2026_7230_MOESM2_ESM.docx]

# Supplementary files 1.

**Table E1**. **Comparison of Characteristics Between Frail and Non-Frail Participants**

| Characteristic | Category | Frailty (N=2,792) n (%) | Non-Frailty (N=10,894) n (%) | P-value |
| --- | --- | --- | --- | --- |
| Sex | Male | 1255 (44.9) | 4757 (43.7) | 0.217 |
|  | Female | 1537 (55.1) | 6137 (56.3) |  |
| Age group | 60-64 years | 927 (33.2) | 3521 (32.3) | 0.221 |
|  | 65-69 years | 734 (26.3) | 3244 (29.8) |  |
|  | 70-74 years | 699 (25.0) | 2523 (23.2) |  |
|  | 75-79 years | 432 (15.5) | 1606 (14.7) |  |
| Geographic Region | Northeast | 414 (14.8) | 1581 (14.5) | <0.001 |
|  | East | 362 (13.0) | 1621 (14.9) |  |
|  | Central | 325 (11.6) | 1637 (15.0) |  |
|  | North | 399 (14.3) | 1566 (14.4) |  |
|  | South | 263 (9.4) | 1689 (15.5) |  |
|  | Northwest | 658 (23.6) | 1260 (11.6) |  |
|  | Southwest | 371 (13.3) | 1540 (14.1) |  |
| Education Level | Primary school or below | 1681 (60.2) | 6388 (58.7) | 0.065 |
|  | Middle school | 973 (34.8) | 3838 (35.2) |  |
|  | High school or above | 138 (4.9) | 668 (6.1) |  |
| Marital Status | Married | 2445 (87.6) | 10093 (92.6) | <0.001 |
|  | Widowed | 235 (8.4) | 520 (4.8) |  |
|  | Single | 33 (1.2) | 120 (1.1) |  |
|  | Divorced | 79 (2.8) | 161 (1.5) |  |
| Living Status | Living alone | 429 (15.4) | 1084 (10.0) | <0.001 |
|  | Not living alone | 2363 (84.6) | 9810 (90.0) |  |
| Household Income | 0-1k | 199 (7.1) | 652 (6.0) | 0.094 |
|  | 1k-5k | 1482 (53.1) | 5736 (52.7) |  |
|  | 5k-10k | 973 (34.8) | 3838 (35.2) |  |
|  | >10k | 138 (4.9) | 668 (6.1) |  |
| Sleep Quality | Very poor | 273 (9.8) | 503 (4.6) | <0.001 |
|  | Poor | 128 (4.6) | 191 (1.8) |  |
|  | Fair | 1196 (42.8) | 3637 (33.4) |  |
|  | Good | 706 (25.3) | 3707 (34.0) |  |
|  | Very good | 489 (17.5) | 2856 (26.2) |  |
| Life Satisfaction | Very dissatisfied | 175 (6.3) | 203 (1.9) | <0.001 |
|  | Dissatisfied | 258 (9.2) | 787 (7.2) |  |
|  | Neutral | 431 (15.4) | 955 (8.8) |  |
|  | Satisfied | 1561 (55.9) | 6814 (62.5) |  |
|  | Very satisfied | 367 (13.1) | 2135 (19.6) |  |
| Smoking Status | Current smoker | 652 (23.4) | 1296 (11.9) | <0.001 |
|  | Non-smoker | 2140 (76.6) | 9598 (88.1) |  |
| Alcohol Use | Current drinker | 692 (24.9) | 1510 (12.7) | <0.001 |
|  | Non-drinker | 2100 (75.1) | 9384 (85.2) |  |
| Physical Activity | Meets WHO guideline | 959 (34.3) | 2909 (26.7) | <0.001 |
|  | Does not meet guideline | 1833 (65.7) | 7985 (73.3) |  |
| Body Mass Index Category | Underweight | 91 (3.3) | 239 (2.2) | 0.001 |
|  | Normal weight | 1126 (40.3) | 4687 (43.0) |  |
|  | Overweight | 1178 (42.2) | 4583 (42.1) |  |
|  | Obese | 395 (14.1) | 1380 (12.7) |  |
| Sedentary Behavior | High (>4 hours/day) | 1093 (39.1) | 2946 (27.0) | <0.001 |
|  | Low (≤4 hours/day) | 1699 (60.9) | 7948 (73.0) |  |
| Relative STS Power | Low | 1162 (41.6) | 4031 (37.0) | <0.001 |
|  | High | 1630 (58.4) | 6863 (63.0) |  |

**Table E2. Associations of the Risk for Frailty and Relative Stand-to-sit Power**

| Types of variables | Crude Model | | Adjusted Model | |
| --- | --- | --- | --- | --- |
|  | OR (95%CI) | *P* Value | OR (95%CI) | *P* Value |
| Categorical variable | 1.20 (1.10, 1.31) | <0.001 | 1.12 (1.01, 1.23) | 0.02 |
| Continuous variable | 1.05 (1.01, 1.09) | 0.01 | 1.08 (1.05, 1.12) | <0.001 |

**Table E3**. Sensitivity analyses additionally adjusting for screen time.

| variables (cut-off value) | OR (95%CI) | *P* Value |
| --- | --- | --- |
| Sedentary behavior(4 hous) | 1.51 (1.37, 1.67) | <0.001 |
| Sedentary behavior(6 hous) | 1.85 (1.59, 2.15) | <0.001 |
| Sedentary behavior(8 hous) | 1.12 (0.86, 1.44) | 0.41 |
| Sedentary behavior(10 hous) | 2.16 (1.36, 3.42) | <0.001 |
| Sedentary behavior(increase 1 hours) | 1.10 (1.07, 1.13) | <0.001 |

**Table E4**. Sensitivity analyses restricted to participants with complete covariates.

| variables (cut-off value) | Crude Model | | Adjusted Model | |
| --- | --- | --- | --- | --- |
|  | OR (95%CI) | *P* Value | OR (95%CI) | *P* Value |
| Sedentary behavior(4 hous) | 1.73 (1.59, 1.89) | <0.001 | 1.59 (1.45, 1.75) | <0.001 |
| Sedentary behavior(6 hous) | 2.24 (1.07, 2.55) | <0.001 | 2.01 (1.75, 2.31) | <0.001 |
| Sedentary behavior(8 hous) | 1.72 (1.37, 2.15) | <0.001 | 1.39 (1.08, 1.76) | 0.02 |
| Sedentary behavior(10 hous) | 2.73 (1.79, 4.13) | <0.001 | 2.53 (1.60, 3.96) | <0.001 |
| Sedentary behavior(increase 1 hours) | 1.15 (1.13, 1.18) | <0.001 | 1.12 (1.09, 1.14) | <0.001 |

**Table E5. Interaction in all data**

|  | **High STS** | **Low STS** | **Effect of low STS within the strata of high SB** |
| --- | --- | --- | --- |
|  | OR [95% CI] | OR [95% CI] | OR [95% CI] |
| **Low SB** | 1 [Reference] | 1.05 [0.94, 1.19] |  |
| **High SB** | 1.48 [1.31, 1.68] | 1.84 [1.6, 2.12] | 1.24 [1.06, 1.45] |
| **Effect of high SB within the strata of low STS** |  | 1.75 [1.51, 2.03] |  |
| **Multiplicative scale** | 1.18 [0.97, 1.43] |  |  |
| **RERI** | 0.31 [0.02, 0.59] |  |  |
| **AP** | 0.17 [0.01, 0.29] |  |  |
| **SI** | 1.57 [1, 2.46] |  |  |

Definition of abbreviations: SB = sedentary behavior; STS = relative stand-to-sit power; RERI = Relative Excess Risk due to Interaction; AP = Attributable Proportion due to Interaction; SI = Synergy Index.

**Table E6. Interaction in rural data**

|  | **High STS** | **Low STS** | **Effect of low STS within the strata of high SB** |
| --- | --- | --- | --- |
|  | OR [95% CI] | OR [95% CI] | OR [95% CI] |
| **Low SB** | 1 [Reference] | 1.01 [0.87, 1.17] |  |
| **High SB** | 1.22 [1.05, 1.43] | 1.83 [1.53, 2.19] | 1.5 [1.23, 1.82] |
| **Effect of high SB within the strata of low STS** |  | 1.82 [1.5, 2.2] |  |
| **Multiplicative scale** | 1.49 [1.17, 1.9] |  |  |
| **RERI** | 0.6 [0.27, 0.95] |  |  |
| **AP** | 0.33 [0.15, 0.46] |  |  |
| **SI** | 3.62 [1.17, 11.17] |  |  |

Definition of abbreviations: SB = sedentary behavior; STS = relative stand-to-sit power; RERI = Relative Excess Risk due to Interaction; AP = Attributable Proportion due to Interaction; SI = Synergy Index.

**Table E7. Interaction in urban data**

|  | **High STS** | **Low STS** | **Effect of low STS within the strata of high SB** |
| --- | --- | --- | --- |
|  | OR [95% CI] | OR [95% CI] | OR [95% CI] |
| **Low SB** | 1 [Reference] | 1.16 [0.95, 1.42] |  |
| **High SB** | 2 [1.62, 2.46] | 1.91 [1.51, 2.42] | 0.95 [0.74, 1.23] |
| **Effect of high SB within the strata of low STS** |  | 1.64 [1.29, 2.09] |  |
| **Multiplicative scale** | 0.82 [0.6, 1.13] |  |  |
| **RERI** | -0.25 [-0.82, 0.29] |  |  |
| **AP** | -0.13 [-0.49, 0.12] |  |  |
| **SI** | 0.78 [0.46, 1.32] |  |  |

Definition of abbreviations: SB = sedentary behavior; STS = relative stand-to-sit power; RERI = Relative Excess Risk due to Interaction; AP = Attributable Proportion due to Interaction; SI = Synergy Index.
